# A survey of Australian women’s digital media usage in pregnancy and labour and birth

**DOI:** 10.1186/s12884-023-06003-8

**Published:** 2023-09-23

**Authors:** Ilyana Mohamed Hussain, Nicki Hartney, Linda Sweet

**Affiliations:** 1 School of Nursing and Midwifery, Deakin University, Victoria, Australia; 2 Western Health, Victoria, Australia; 3 Centre for Quality and Patient Safety Research, Institute for Health Transformation, Western Health Partnership, 221 Burwood Highway, Burwood, VIC 3125 Australia

**Keywords:** Digital media, Pregnancy, Labour and birth, Social media, Information-seeking, Reassurance

## Abstract

**Background:**

Given the rapid growth of digital media resources, it is worth exploring childbearing women’s use of digital media to address their information needs. The aim of this study was to explore the use of digital media during pregnancy and birth in the local population of Western Victorian women in Melbourne, Australia.

**Methods:**

A descriptive exploratory approach was used. An online survey consisted of both quantitative and qualitative questions to identify and measure digital media use in pregnancy and the birthing period. Descriptive statistics and Pearson Chi-square test were used to analyse the quantitative data, while content analysis was used to analyse the qualitative data.

**Results:**

Digital media has become an integral part of the experience in pregnancy with increasing growth of digital media in labour. The most used medium for digital media use was pregnancy applications, followed by websites, social media, YouTube, podcasts, online discussion forums and lastly, labour applications. Information seeking was the main reason for using digital media, and two main themes emerged from the qualitative data; ‘connection with others for social support and reassurance’ and ‘information seeking and providing to assist decision making and providing reassurance’.

**Conclusion:**

This study highlights the need for future midwifery practice to include digital media sources in antenatal education and care. There is a need for healthcare institutions to improve digital media technology to meet the needs of women. This is crucial as digital media is constantly evolving, and as healthcare providers, we need to integrate digital media with healthcare services.

**Supplementary Information:**

The online version contains supplementary material available at 10.1186/s12884-023-06003-8.

## Introduction

Traditionally, health education in pregnancy is provided face-to-face during antenatal appointments and with pamphlets, audio-visual, and mass media campaigns [1, 2]. Since the emergence of the internet, digital media has provided additional advice and support for many women [2, 3]. Within the past decade, the internet has become a popular source of information for pregnant women [4]. The ease of access, convenience, minimal cost, and timely information have driven the increased use of digital media [4, 5]. Dissatisfaction with information received during pregnancy care and the ease of access to digital media has resulted in pregnant women searching the internet for answers to their questions about pregnancy, labour, and birth [6]. The Internet is valuable for women with rare conditions (e.g. fetal anomalies) where general pregnancy information may not provide relevant content [7]. Sharing stories online enables women to connect emotionally and compare their responses to others, validate feelings, and not feel completely isolated knowing others have gone through similar situations [6, 7].

Pregnancy is a time when women are motivated to make health behaviour changes and are exposed to health information as part of their routine antenatal care [8]. Health information-seeking behaviour is a key coping strategy promoting healthy activities and adjusting psychosocially to illness [9]. Healthcare professionals provide antenatal care, health education, and health promotion. However, research has found that women were not satisfied with the information provided by their healthcare professionals and were turning to digital media to enhance their health literacy [10]. However, this form of self-help is not without consequences. Vamos et al. [11] found increased anxiety levels in women searching and receiving information online. Whilst digital media offers a solution for pregnant women, it also may arouse feelings of anxiety, self-responsibility, and blame [12]. The emergence of pregnant women to have responsibilisation can be both burdensome and empowering [13].

A previous study by Lupton and Pedersen [14] highlighted the increasing number of Australian women turning to pregnancy and parenting applications (apps) as a source of information, self-assurance, and support during the pregnancy and early parenting period. As women increasingly turn towards multiple forms of digital media and not just apps, there is limited research on what digital media women use and why. However, an emerging body of literature looks to understand the sociocultural impact and why pregnancy apps are at the frontier for pregnancy information and commercialisation for women during that period [12, 13].

Pregnancy is an opportunistic time for health promotion as women are keen to explore what happens to them and how to prepare for birth [15]. Apps and social media sites are a new way of how women project their responsibilisation towards their health and the means to improve and monitor their pregnancy and the progress of the unborn child [16]. Decisions made by women during this period are influenced by the information received from digital media [2, 17, 18]. Digital media use has been shown to help women with decision-making during pregnancy and birth, share experiences, and develop confidence when speaking to healthcare professionals [3, 10, 19].

Limited studies focus on digital media use and purpose during labour and birth. Recent research [20] hasfound that women use apps to time their contractions before presenting to the hospital for labour assessment. A Western Australian study identified that women valued the use of mobile phones within the hour post birth primarily to notify family and friends [21]. This showed that women valued digital media during and immediately post labour and birth. It is, therefore, worthy to explore the use of digital media not only in pregnancy but also during labour and birth.

This research aimed to explore the digital media use specifically related to pregnancy, and labour and birth, within the local population of Western Victorian women in Melbourne, Australia. For this study, pregnancy, and labour and birth related digital media encompass mobile applications (apps), social media platforms (Facebook, Instagram, TikTok), online search engines and websites, YouTube, and Podcasts.

## Methods

### Study design and ethics

A cross-sectional descriptive exploratory design using an online survey hosted on the Qualtrics platform was used. The study was granted ethical approval by the Low-Risk Human Ethics Committee at Western Health (HREC/21/WH/73,249). The study setting was a single tertiary hospital in metropolitan Melbourne that provides maternity services for over 6500 pregnant women per year.

### Inclusion and exclusion criteria

The inclusion criteria included women aged 18 to 45 years who were either pregnant or had a baby at the study site in the previous two years. Primiparous and multiparous women were eligible, and women pregnant at any gestation were included. A basic understanding of English was required to participate, as the study was conducted in English only. Potential participants were excluded if they did not declare they were over 18 years or had not read the Plain Language Statement (PLS). Once commencing the survey, participants who provided an age of less than 18 or more than 45 were excluded and sent to the end of the survey.

### Recruitment

Non-probability sampling was used in this study. Three convenience sampling approaches were used to recruit women. Firstly, was face-to-face recruitment in the antenatal clinics by the first author, second was placing posters at different locations within the hospital and providing flyers to women attending for pregnancy care, and lastly, a targeted advert was promoted on the hospitals’ Facebook page. A Quick Response (QR) code and an online link to the survey were included on the posters, flyers, and Facebook advertisements.

Potential participants were asked to read the PLS on the survey landing page. The PLS consisted of information about the purpose of the study, what was involved if they chose to participate, the risks and benefits of participating, and how their rights and interests were protected. The PLS clearly stated that participation in the study was voluntary, all data would be anonymous, and participating or not would not affect the care they received in the hospital. Participants were required to declare they had read the PLS and were over 18 of age and consent to progress to the survey.

### Data collection tool

The questionnaire was adapted from the tool used by Lupton and Pederson [14] with permission. As Lupton and Pederson [14] only explored app use during pregnancy, additional questions were added to accommodate the other forms of digital media and questions related to digital media use during labour and birth. Demographic questions included age, marital status, occupation, education level, and gestation or timeframe since the last birth. No identifiable information was collected in the survey. There were 12 demographic and screening questions, including identifying which forms of digital media the participant used. There were nine questions about app use in pregnancy and nine for app use in labour and birth. There were six questions about social media use in pregnancy and six about social media use in labour and birth. There were five questions about YouTube and video Blog use, four questions about website use, four questions about podcast use, and three questions about discussion groups. Skip logic was used to route participants towards specific questions as they progressed through the [22]survey. For example, if a participant responded that they used apps and Facebook during pregnancy only, skip logic only displayed the questions related to these forms of digital media. The questionnaire mainly consisted of closed-ended questions and Likert scales. There were a few open-ended questions to allow participants to provide additional perspectives. Due to the voluntary nature of the survey, participants could exit the survey at any time. The survey was set for single response submission without the capacity of changing or updating responses post submission. Participants were however, able to return to previous questions to change their responses prior to submission. The tool was pilot tested by the research team prior to data collection to check flow and content.

### Data analysis

Data were downloaded and interrogated for completeness. Incomplete cases were removed prior to analysis. Frequencies, percentages, and means were used to describe the quantitative data [23]. The data was then coded into two categorical variables for age (equal or less than 30 years and more than 30 years old), education (with and without university education), place of birth (born in Australia or overseas), and parity (primiparous and multiparous). Pearson chi squared was used to compare these variables against types of digital media and quantity of apps. Chi-square tests were used to determine if the relationship between two variables were statistically significant [23]. Inductive content analysis methods [24, 25] were used to analyse the data from the open-ended questions. The first author identified codes and categories and mapped these out in Microsoft Excel [26]. The research team then checked the coding and categorisation of the qualitative responses to enhance rigour [27]. Illustrative quotes were identified to demonstrate the content analysis findings.

## Results

Data collection occurred over four months in 2021. There were 182 participants that accessed the survey. Six were under 18 years of age, and five were above the age of 45 years, so skip logic sent them to the end of the survey. A further, 21 women consented but did not answer sufficient questions, all of whom were also removed. Analysis was conducted on 142 completed surveys.

The demographic details of the participants are shown in Table 1 of the supplementary file. Of the 142 respondents, approximately half (49%) were currently pregnant, and most of these (62%) were 25–36 weeks pregnant. Most participants (77%) were multiparous, and most (68%) were aged between 25 and 34 years of age. Most respondents identified as being born in Australia (71%), with English as the primary language spoken at home (91%). Women were mainly well educated, with 56% having completed a university degree. All participants had regular access to the internet either via home modem or their mobile phones or tablets.

### Digital media use in pregnancy and birth

**Fig. 1:**
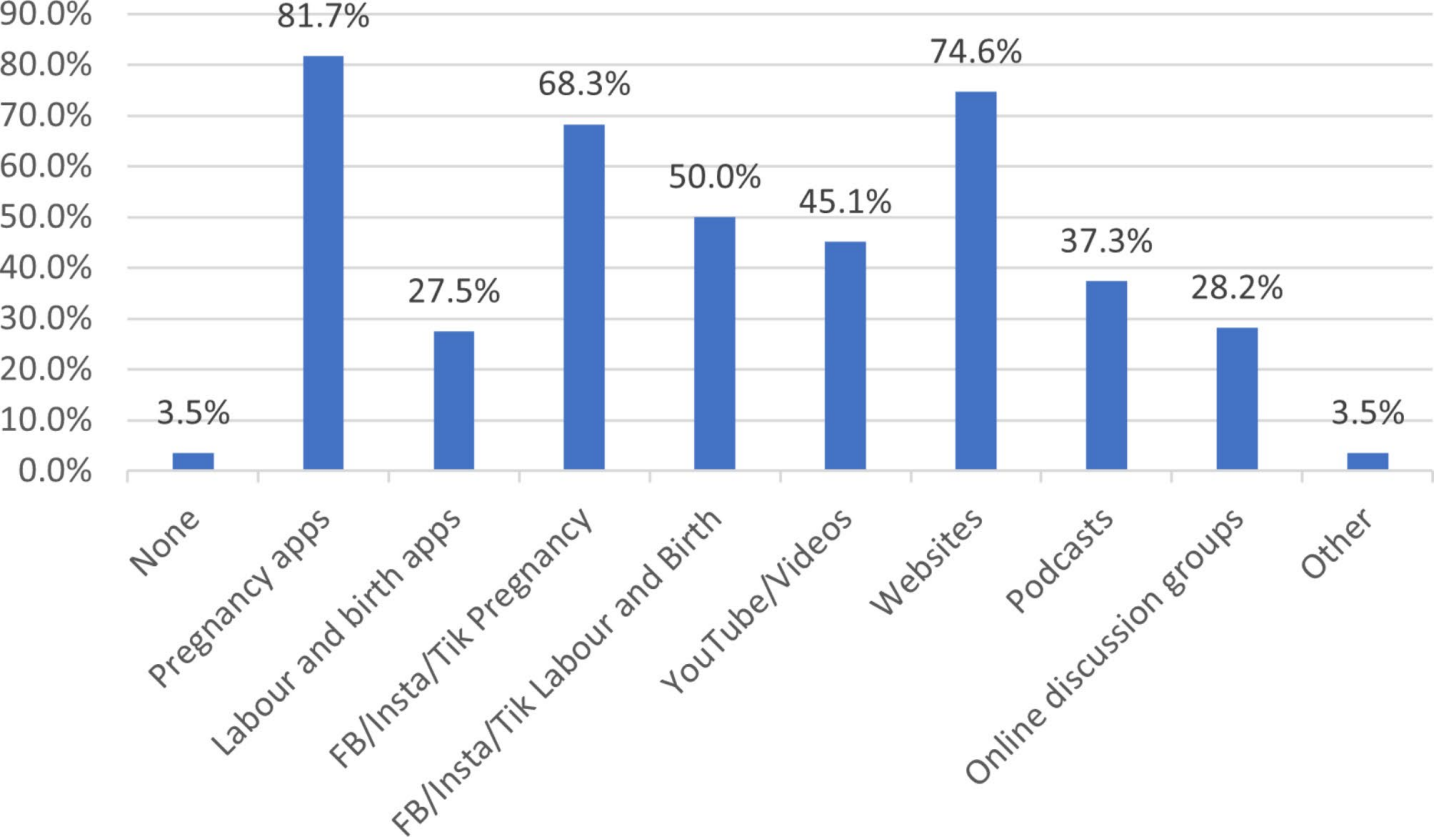
Digital media use in pregnancy and birth. FB = Facebook, Insta = Instagram, Tik = TikTok

As seen in Fig. 1, women mostly used pregnancy apps, followed by websites, then social media sites such as Facebook, Instagram or TikTok as their digital media sources.

### Pregnancy apps usage

The type of Pregnancy app and how and why they were used is shown in Table 2 of the supplementary file. Popular pregnancy apps included ‘BabyCentre’, ‘What to expect’, and ‘Pregnancy+’. A third of the women said they used the apps every week or so, or a few times a week and they were mainly used to get information on their baby’s development and information about changes in their body during pregnancy. Women felt that the apps were giving helpful information (n = 72), enabling them to monitor their baby’s development (n = 60), reassurance (n = 48), and changes to their own body (n = 44). There were women who felt that pregnancy apps were not useful for them due to getting tired of using them (n = 8) or making them feel too anxious or worried (n = 3). Many participants had been recommended digital media by their family or friends (n = 48). Most women did not check where the information from the app came from (n = 78) and they were not concerned about how personal information or images might be used by the app owners (n = 35).

### Labour and birth apps usage

The type of labour and birth apps and how and why they were used is shown in Table 3 of the supplementary file. There were only 39 (27.5%) respondents that used labour and birth apps compared to 116 (81.7%) using pregnancy apps as seen in Fig. 1. Most women that used the labour and birth apps opted for the ‘Contraction timer’ app (n = 16) and ’Spotify’ (n = 15). Women started using the labour and birth apps when they were less than 12 weeks pregnant (n = 12) and only 4 women when in labour. Women were mainly timing contractions in labour (n = 22), seeking information on what to expect in labour and birth (n = 15), and what to pack for the hospital (n = 11). Most women who used labour and birth apps found them to be useful (85.3%). The minority of women felt tired using the apps (n = 3) and thus felt that it was not useful for them. Similarly with pregnancy apps, most women chose the apps based on recommendations by family or friends (30%), followed by cost (22%). Most women, similarly, with pregnancy apps, did not check where information was obtained for the apps (74.4%) and felt unconcerned about personal data being used as the apps did not require any personal information being uploaded (35.9%).

### Social media usage

Table 4 of the supplementary file shows which social media, such as Facebook, Instagram, or TikTok that women accessed during pregnancy and labour and birth, and how and why they used it. There were 97 women who used Facebook, Instagram, or TikTok during pregnancy, whilst 71 women used it during labour and birth. On average, women were spending a few times a week on social media. Women were using social media for information about their pregnancy (n = 50), to connect with other mothers to be (n = 50), to look for products to help with pregnancy aliments (n = 44), update family and friends with progress of pregnancy (n = 43), pregnancy announcement (n = 41), and to post pregnancy-related information (n = 38). Meanwhile, most women used social media to read or watch people’s labour and birth stories (n = 44), followed by birth announcement (n = 26), and connect with women who had similar experiences (n = 23). Nearly half of the women were not worried about where the information for pregnancy or labour and birth that others posted came from. Most women were also not concerned about how personal information or image was used in the social media.

### YouTube and Video usage

The type of videos and YouTube or other videos and how and why they were used is shown in Table 5 of the supplementary file. A total of 64 women used YouTube and Videos during pregnancy and labour and birth. Topics of interests during pregnancy consists of ‘what to expect in pregnancy’ (n = 28), ‘pregnancy exercises’ (n = 25), ‘fetus development’ (n = 24), ‘product reviews’ (n = 22), and ‘tips for pregnant women’ (n = 20). Meanwhile videos related to labour and birth are ‘what to pack’ (n = 24), ‘labour and birth videos’ (n = 22), ‘labour breathing techniques’ (n = 20), ‘labour and birth vlogs or stories’ (n = 19), ‘one born every minute’ (n = 18), and ‘what to expect after birth’ (n = 16). There was no average time spent viewing YouTube or other videos, it varied from every month or so (n = 12), every week or so (n = 10), only once or twice (n = 10), daily (n = 8), and a few times a week (n = 7). Most women did not feel worried about the content provided.

### Website usage

The type of websites, how and why they were used is shown in Table 6 of the supplementary file. The second most used digital media is websites with 106 women choosing to access these during pregnancy and labour and birth. The most accessed websites were, ‘What to expect’ (n = 53), ‘Better health’ (n = 40), and ‘Babycenter’ (n = 36). On average, women would access websites every month or so (n = 24), or every week or so (n = 24). Most women did not worry where the information was obtained from the websites accessed.

### Podcast and online discussion group usage

The podcasts listened to and participation on online discussion groups as well as how and why they were used is shown on Table 7 of the supplementary file. There were 53 women that listened to podcast or engaged in online discussion groups in their pregnancy. ‘Australian birth stories’ was ranked highest with n = 26 women listening to it, followed by ‘Baby and beyond’ (n = 9), and ‘HelloBump’ (n = 8). Nearly half (n = 29) listened to pregnancy related podcasts daily. Most of the women were not concerned with where the information came from for the podcast content.

Women who engaged with pregnancy related online discussion groups (n = 40), were reading about what other people posted (n = 36) or posted about their own experiences (n = 21). Some felt comfortable posting questions (n = 24), whilst others gave advice to others (n = 15). participation in discussion groups were every month or so (n = 11), only once or twice (n = 9), or every week or so (n = 8).

### Statistical analysis

Women less than or equal to 30 years of age used Facebook for birthing information p = .007 and You Tube for pregnancy and birth information p < .001 more than women over 30 years of age. Australian born women were statistically significantly more likely to use Pregnancy apps (p < .001), Facebook for pregnancy information (p < .001), and podcasts for pregnancy and birth information (p = .048) than women not born in Australia. Similarly, women born in Australia were more likely to use more pregnancy apps than women not born in Australia (p = .016). Those women with a university qualification were statistically more likely to use websites about pregnancy than women not having a university degree (p = .032).

### Qualitative data

Content analyses was conducted on the open text questions, and two main themes were constructed; ‘social connection and support’, and ‘information seeking and providing’. Within these two themes there are five sub-themes.

#### Social connection and support

Most women described using digital media in pregnancy to connect with others, primarily as a form of peer support and for social support and reassurance from family and friends.

#### Seeking peer support

Most women described digital media as a means of connection with others as it allowed them to normalise their experience in pregnancy. For example, one woman said, *‘it gave me peace of mind that my situation was somewhat normal’*. Given the study was conducted during the COVID-19 pandemic, peer support allowed respondents to feel connected when they did not have any other social interactions. One woman explained, *‘Being pregnant during COVID was hard, there wasn’t really any other way to get support.’* Similarly, another wrote, *‘Isolation, no face-to-face support groups’*, and another, *‘I was pregnant during the lockdown, so it helped me feel supported by other mums.’*

Many women felt that digital media was an opportunity to establish connections with other pregnant women. For example, one woman wrote, *‘[they] allowed me to join support groups for other expectant mothers due around the same time, allowing for advice and information to be shared’.* For one woman, *‘I was looking for women to connect with who were experiencing the same things … I’ve made some lifelong friends in all of them’.*

However, for a few respondents, connecting with other women was a negative experience, and they removed themselves from the digital media. One participant explained,

*I actually ended up removing myself from groups on Facebook that connected me to other mums, as it just made me too anxious with all that could go wrong, and the ‘negative Nancy’ comments. [I] Felt a lot better once I removed [myself].*

While another expressed, ‘*sometimes it feels as though people want to share their horror stories more than provide helpful information’.*

#### Connecting with family and friends

Most women felt that digital media gave them the platform to update family and friends on their pregnancy and birth announcements. For example, *‘Updating family and friends, following experts to get updates from them’*, and another wrote, *‘I was also able to connect with family and friends and keep everyone updated on my pregnancy’*, and *‘Sharing with friends and family birth announcement’.*

Digital media was for some women it is especially important due to distance. One woman wrote, ‘*to connect to others like my family who is far away for them to experience my pregnancy*’ and another, ‘*connect with family overseas’.* However, some expressed that they have *‘very few friends with babies’* and *‘didn’t want to feel alone’* as a reason to *‘finding other mums to be, making friends online and being there throughout our pregnancies’*.

#### Information seeking and providing

Most women identified how using digital media in pregnancy provided them *‘ease of access’* and ‘*accessibility, [that was] not invasive, informative and flexible’*. One woman described how pregnancy information would *‘pop up’* in her digital media as a result of the searches she had done:

*Nice to see Instagram posts pop up in my search feed, and I could read mum blogs/posts. I don’t rely on Instagram for actual pregnancy information (I would always speak to my midwife team for actual information). I understand that a lot of information given on social media is not fact checked/may not be accurate.*

Some women felt the need for pregnancy information specifically tailored to their needs, such as, *‘As a type 1 diabetic, it was good to be a part of a Facebook page run by a midwife/endo nurse and someone I trust. I know her off digital also’.* Similarly, others wrote, *‘to chat with people sharing similar pregnancy issues (hyperemesis and placenta previa)’*, and *‘managing pregnancy issues, such as heartburn, haemorrhoids, restless legs, nausea.’*

Some women expressed the importance of having access to information provided by healthcare professionals. One woman explained, ‘*information provided by the midwife was good as she gave facts and opinions without trying to push any views or scare people listening’*. Another woman explained, ‘*I follow some really great evidence-based health professionals who share invaluable information (e.g., Birth with Beth, Physio Laura)’* and another wrote, *‘I happened to find some great health professionals on Instagram and valued the evidence-based information they generously shared’.*

#### Desire for information about pregnancy, labour and birth

The infrequent antenatal visits, especially due to the COVID pandemic, appeared to influence the participants digital media use. One woman wrote, *‘During COVID, didn’t have as much access to health professionals’*, and another, *‘It was great to have the information at my fingertips as the midwife appointments were not regular to ask questions.’* This was common, as one woman explained, *‘Limited face-to-face appointments due to COVID’.*

For one of the women, she *‘needed more information and felt compelled to immerse myself in content related to what I was going through’*, while another felt the need for *‘up to date information as opposed to older content published in print and books.’* Most women expressed *‘wanting to learn more about my pregnancy’*, with one explaining, ‘*Everything is digital now. It’s how we collect information and view things. I read some books, but it’s helpful to see visually what to expect’*, demonstrating how they were turning to digital media as a source of information.

However, some expressed concerns about the information found through digital media. Reliability was raised, with one woman writing, *‘I want to be able to understand what reliable research has been done about various decisions I have to make, so I can make informed decisions. But so much info online is overly defensive/fearmongering/not supported by any rationale’.* Similarly, another woman wrote, *‘Video blogs [were the] only way of education, so were great to a point. Again, [they] got too deep and started to make us feel more panicked about what could go wrong, so I stopped watching’.*

#### Contributing for others - a sense of giving back

Some women felt that digital media allowed them to share their own experiences as well as ask questions. One woman wrote, *‘[to have] Ongoing discussions that were relating to pregnancy that I could join in and contribute to and ask follow-up questions’*, and another, *‘I really just used Facebook to make jokes about all the weird changes my body was going through and if other mums experienced these things’.*

#### Reassurance from hearing other peoples’ experiences

Listening to other people’s experiences encouraged positive emotions in most women. One woman explained, *‘So inspiring listening to other incredible women’s birthing experiences, helped put me in a positive headspace prior to birthing’*, and similarly, *‘I love listening to other women’s birth stories. it helped me enjoy my pregnancy and prepare for my homebirth.’* Sharing experiences was soothing for some women, *‘Listening to others’ experiences helped put my mind at ease’*, and *‘I read and watched so many births before going into labour to help get me in a good headspace.’*

Hearing other women’s stories allowed participants to gain knowledge for their decision -making. One woman explained, *‘exposure to more stories and resources made me feel more prepared for the possibilities of labour/birth and education is the key to making informed choices’*, and similarly, ‘…*decision to try for a VBAC [vaginal birth after caesarean]’*, and ‘*exploring different perspective relating to birth. Making sure I was able to experience a feminist pregnancy in which I was fully empowered about the choices I was making’.*

## Discussion

This study explored different types of digital media used in pregnancy and the labour and birth period and shows how digital media has become an integral part of the experience in pregnancy for many women who completed this survey. The findings from this study show women are accessing different sources of information to meet their needs. We found that pregnancy apps were the most popular choice of digital media, followed by websites, social media, YouTube, podcasts, online discussion groups, and lastly labour apps. Most women cited the reasons for choosing digital media was through recommendations from friends, family, and/or healthcare professionals.

The women who accessed digital media cited information-seeking as one of the main reasons for using it. this finding concurs with other studies [2, 3], which also found women use digital media to gain knowledge and enhance their understanding. Most women from this study described connection with others through digital media as a means of social support and reassurance. This is also seen in previous studies [5, 15]. In comparison, a study [10] conducted in Sweden showed that 65.6% of women felt worried after reading pregnancy-related information online, and some women expressed this sentiment in our study. One of our participants removed herself from a Facebook group due to feelings of anxiety.

As seen in Bjelke et al. [10] the number of women attending antenatal parental education has decreased in the last five years as more women sought information and social interaction online due to the increasing resources available digitally. Similarly, research has found women were turning to the internet due to inadequate information provided by health professionals or due to limited opportunities for discussion from fewer antenatal clinic appointments [1, 3]. This is evident in our study, as women described not having as much access to health information during antenatal clinic appointments as they would like, but also because there were no antenatal classes provided by the health service.

There was no statistical significance in the aspect of usage of digital media found between primiparous women and multiparous women. Australian-born women were statistically significantly more likely to use pregnancy apps, Facebook for pregnancy information, and podcasts for pregnancy and birth information than those not born in Australia. Sociodemographic factors such as higher level of education and age did show a correlation for increased use of digital media. Those participants with a university degree were statistically significantly more likely to use digital media (Pregnancy apps, Facebook, and podcast) than those who did not have a university degree.

It was beyond the scope of this study to assess the accuracy of the information used. However, women were asked if they felt concerned about personal information that might be used by developers and if they checked where the information came from. Most women in this study were not concerned and did not feel the need to check where the source of information came from. These findings are consistent with Lagan et al. [3] where 75% of respondents did not check the credibility of information obtained from social media. However, women in this study did identify the preference for evidence-based information and that health care providers direct them to trustworthy sources.

Our findings are consistent with the concepts of responsibilization [13, 28] and the lay expert [7, 13, 16]. Women in this study were not afforded any specific antenatal education classes by the health service (beyond their antenatal appointments). They were recommended to seek this from an external provider as a cost to the user. Moreover, this study occurred during the COVID-19 pandemic when many services were reduced or restricted, including in-person antenatal appointments. Such situations unintentionally placed a level of responsibilization on women to seek the information they desired [13, 16, 28], but also become the lay expert as women were able to access information in a timely manner [7]. Furthermore, it is clear that pregnancy related information in the digital age has created a level of lay expert, where women can ‘cherry pick’ information, advice, and personal experience that resonates with them that may not be evidence based [7, 13, 16]. Participants in our study clearly showed they did not check the accuracy of the information they accessed; a finding consistent with other studies [7, 13]. No specific details were sought about participants’ individual pregnancy concerns, so we are unable to link information seeking to pregnancy related issues as has been done in previous studies [7, 13, 28]. However, it is clear from our findings that women sought to educate themselves about pregnancy, labour and birth beyond what they received through standard maternity care.

Our findings can inform future midwifery practice in directing women towards appropriate sources of information. As seen in this study, information-seeking behaviours can evoke a sense of empowerment for women, and as health professionals, we should recognise the need for quality, evidence-based resources for women to access.

### Study strengths and limitations

The strength of this study lies in investigating multiple forms of digital media focused on the pregnancy period but also the labour and birth period. The percentage of Australian to ethnic background respondents reflected the hospital’s multicultural background and can be considered a strength of this study. The combination of both quantitative and qualitative questions has enabled the exploration of women’s subjective experience. Data from the closed-ended questions enabled us to understand the different types of digital media used and the motivations for using certain digital media. Respondents were able to express different views by entering ‘freetext’ options.

Despite the strengths, there were also limitations. As this study was conducted in a single large tertiary hospital, the findings may not reflect the experiences of Australian women in other areas. Furthermore, the study was over-represented by women who had completed higher education, which does not reflect the community in which it was conducted. ‘Response burden’ [27] was also seen in this study, due to the length of the survey, many respondents commenced but did not complete the study.

### Implications for practice and/or policy

This study highlights the need for maternity service providers to be aware of the evolving nature of digital media, the integration of digital media with healthcare services, and what needs it is addressing for women. Future research may provide some direction to organisations that support and establish digital media resources for women during the different stages of pregnancy and early parenting. Exploration of effective ways for health professionals to provide information to women, sharing of information between women and health professionals, and how women can be better supported for their information needs could be investigated in the future. Additional research on the impact of digital media use and the potential negative consequences for pregnant women could be studied.

## Conclusions

The survey reported here provides insights into how Australian women used digital media during pregnancy and during their labour and birth. This is a unique study as it included different types of digital media. Our findings highlight how important digital media has become as a resource for women to access to meet their information and social networking needs during pregnancy, labour, and birth. Women also expressed their desire for the healthcare provider to be able to direct and assist them in obtaining evidence -based and recommended information.

### Electronic supplementary material

Below is the link to the electronic supplementary material.

Supplementary Material 1: The supplementary file contains the Tables 1–7

## Data Availability

All data generated or analysed during this study are included in this published article.

## Abbreviations

Apps: Applications
