# Supplementary material for: A survey of Australian women’s digital media usage in pregnancy and labour and birth
Source: BMC Pregnancy Childbirth. 2023 Sep 23;23:688. doi: 10.1186/s12884-023-06003-8 (PMC10517456; doi:10.1186/s12884-023-06003-8)
Supplement: Supplementary file 1 — Supplementary Material 1: The supplementary file contains the Tables 1–7 [file 12884_2023_6003_MOESM1_ESM.docx]

# Tables

Table 1: Participant Characteristics

| Characteristics  (N=142 unless otherwise indicated) | Variables | n (%) |
| --- | --- | --- |
| Currently pregnant and receiving care at [redacted for peer review] | No | 73 (51.4%) |
|  | Yes | 69 (48.6%) |
| Current gestation (for those pregnant)  (n=69) | 25-36 weeks | 43 (62.3%) |
|  | 37 or more weeks | 18 (26.1%) |
|  | 13-24 weeks | 8 (11.6%) |
|  | 12 weeks or less | 0 (0%) |
| Given birth at [redacted for peer review] in the last two years | Yes | 73 (51.4%) |
|  | No | 69 (48.6%) |
| Number of children | 1 | 66 (46.5%) |
|  | 2 | 43 (30.3%) |
|  | None but currently pregnant | 33 (23.2%) |
|  | 3 or more | 0 (0%) |
| Current age (years) | 25-30 | 54 (38%) |
|  | 31-34 | 43 (30.3%) |
|  | 35-40 | 35 (24.6%) |
|  | 18-24 | 6 (4.2%) |
|  | 41 or more | 4 (2.8%) |
| Country of birth  (n=134) | Australia | 101 (71.1%) |
|  | Other | 9 (6.3%) |
|  | India | 5 (3.5%) |
|  | Philippines | 5 (3.5%) |
|  | New Zealand | 3 (2.1%) |
|  | Pakistan | 3 (2.1%) |
|  | United Kingdom | 3 (2.1%) |
|  | Vietnam | 3 (2.1%) |
|  | Indonesia | 2 (1.4%) |
| Primary language spoken at home | English | 129 (90.8%) |
|  | Other | 13 (9.2%) |
| Highest level of education | Completed a university degree | 79 (55.6%) |
|  | Technical and Further education or apprenticeship training | 21 (14.8%) |
|  | Completed final year of high school (Year 12) | 20 (14.1%) |
|  | Some university education | 13 (9.2%) |
|  | Did not complete the final year of high school (Year 12) | 9 (6.3%) |
| Type of internet they had regular access to | Both internet at home and on a mobile phone or tablet device | 137 (96.5%) |
|  | Internet access only via a mobile phone or tablet device | 3 (2.1%) |
|  | Internet access only via home modem | 2 (1.4%) |
|  | No personal internet access – rely on free Wi-Fi (such as at libraries, shopping centres, or family and friends) | 0 (0%) |

Table 2: Pregnancy App Usage (N= 116)

| Characteristic | Variable | n (%) |
| --- | --- | --- |
| Pregnancy apps used (could select all that apply) | BabyCentre | 53 (45.7%) |
|  | Pregnancy+ | 51 (44.0%) |
|  | What to expect pregnancy & baby tracker | 50 (43.1%) |
|  | Ovia | 26 (22.4%) |
|  | Other | 18 (15.5%) |
|  | Pregnancy App | 10 (8.6%) |
|  | Mind the bump | 10 (8.6%) |
|  | Sprout | 9 (7.7%) |
|  | Pelvic Floor | 5 (4.3%) |
|  | Peanut | 5 (4.3%) |
|  | My pregnancy & baby today | 5 (4.3%) |
|  | Baby2Body | 3 (2.6%) |
|  | Totally pregnant | 2 (1.7%) |
|  | Full Term | 0 (0%) |
| Average use | Every week or so | 40 (34.5%) |
|  | A few times a week | 33 (28.4%) |
|  | Daily | 22 (18.9%) |
|  | Every month or so | 13 (11.2%) |
|  | Only once or twice | 5 (4.3%) |
|  | Not sure/can't remember | 2 (1.7%) |
| Purpose of using App | For information about my baby's development | 112 (96.6%) |
|  | For information about changes in my body related to pregnancy | 87 (75.0%) |
|  | For online discussions with other pregnant women | 38 (32.8%) |
|  | For tracking aspects of my body during pregnancy, such as my weight gain or my diet | 37 (31.9%) |
|  | For keeping/tracking information about my medical appointments, medical test results etc | 18 (15.5%) |
|  | For tracking aspects of my baby, like its heart rate | 16 (13.8%) |
|  | For keeping a pregnancy journal | 15 (12.9%) |
|  | For uploading and storing photos/videos of myself while pregnant | 12 (10.3%) |
|  | For uploading and storing ultrasound images of my baby | 5 (4.3%) |
|  | Other | 3 (2.6%) |
|  | Not sure/can't remember | 0 (0.0%) |
| What was useful about them? | They provided helpful information | 72 (62.1%) |
|  | They helped me monitor my baby's development | 60 (51.7%) |
|  | They gave me reassurance | 48 (41.4%) |
|  | They helped me monitor changes in my own body | 44 (37.9%) |
|  | They helped me connect with other pregnant women | 31 (26.7%) |
|  | They helped me share information about my pregnancy with friends and family | 23 (19.8%) |
|  | They helped me keep track of my medical appointments and records | 9 (7.8%) |
|  | They helped me store photos or videos of my changing body | 7 (6.0%) |
|  | They helped me store ultrasound images | 3 (2.6%) |
|  | Other | 3 (2.6%) |
|  | Not sure/can't remember | 2 (1.7%) |
| Why weren't the pregnancy apps useful for you | I got tired of using them | 8 (6.9%) |
|  | They made me feel too anxious or worried | 3 (2.6%) |
|  | The information was not relevant to me | 1 (0.9%) |
|  | The information provided was inaccurate | 1 (0.9%) |
|  | Not sure/can't remember | 1 (0.9%) |
|  | I don't have enough time to use them | 0 (0.0%) |
| What influenced you to choose these apps? | Recommendation by family or friend | 48 (41.4%) |
|  | Cost | 44 (37.9%) |
|  | Operating system | 27 (23.3%) |
|  | Other | 18 (15.5%) |
|  | Recommendation by health care provider | 9 (7.8%) |
|  | Language used | 7 (6.0%) |
| Did you ever check where the pregnancy app developers obtained the information they used in the app? | No | 78 (60.3%) |
|  | Not sure/can't remember | 15 (12.9%) |
|  | Yes, for all the apps I used | 9 (7.8%) |
|  | Yes, for at least one of the apps I used | 7 (6.0%) |
| Have you ever been concerned about how your personal information/images might be used by the app developers when you use these pregnancy apps? | No, the apps I use don't involve me uploading personal information or images | 37 (31.9%) |
|  | No, I'm not at all concerned | 35 (30.1%) |
|  | Yes, I'm somewhat concerned | 18 (15.5%) |
|  | Don't know/not sure | 15 (12.9%) |
|  | Yes, I'm very concerned | 4 (3.4%) |

Table 3: Labour and birth app usage (N=39)

| Characteristic | Variable | n (%) |
| --- | --- | --- |
| Labour and birth apps used (could select all that apply) (N=39) | Contraction timer | 16 (41.0%) |
|  | Spotify | 15 (38.5%) |
|  | Baby + | 7 (17.9%) |
|  | Labor signs | 5 (12.8%) |
|  | Other | 5 (12.8%) |
|  | Baby Pics | 4 (10.3%) |
|  | Full term | 3 (7.7%) |
|  | GentleBirth hypnobirthing | 3 (7.7%) |
|  | Freya | 3 (7.7%) |
|  | Birth Announcements | 2 (5.1%) |
|  | TMSoft white noise | 2 (5.1%) |
|  | AMMA labour & contraction | 0 (0.0%) |
| When did you start using the Labour and birth apps | Less than 12 weeks pregnant | 12 (30.8%) |
|  | 25-36 weeks pregnant | 8 (20.5%) |
|  | 13-24 weeks pregnant | 6 (15.4%) |
|  | 37 weeks or more | 5 (12.8%) |
|  | When in labour | 4 (10.3%) |
| What did you use the Labour and birth apps for? | Time contractions in labour | 22 (56.4%) |
|  | What to expect for labour and birth | 15 (38.5%) |
|  | Get advice when to go to hospital | 11 (28.2%) |
|  | Information about what to pack and bring to hospital | 11 (28.2%) |
|  | For interacting with other parents | 6 (15.4%) |
|  | Update family and friends about current labour status | 5 (12.8%) |
|  | Keep in contact with family or friends | 5 (12.8%) |
|  | Spread word about birth of child | 3 (7.7%) |
|  | Uploading and storing photos and videos of child | 3 (7.7%) |
|  | For sharing information and images about my child on social media | 1 (2.6%) |
|  | Not sure/can't remember | 1 (2.6%) |
|  | Other | 1 (2.6%) |
| Were Labour and birth apps useful | Yes | 29 (74.4%) |
|  | No | 5 (12.8%) |
| What did you find useful about Labour and birth apps | Provided useful information about what to expect for labour and birth | 17 (43.6%) |
|  | Monitor contraction pattern | 17 (43.6%) |
|  | Gave me information to make me feel prepared for labour and birth | 13 (33.3%) |
|  | Pack for my hospital bags | 10 (25.6%) |
|  | Connect with other mothers | 4 (10.3%) |
|  | Know when it is time to call the midwife | 3 (7.7%) |
|  | Share information about the birth of bub to family and friends | 3 (7.7%) |
|  | Store photos or videos of bub | 2 (5.1%) |
|  | Fill in the blanks | 9 (2.3%) |
|  | Not sure/can't remember | 1 (2.7%) |
|  | Other | 1 (2.7%) |
| Why the Labour and birth apps were not useful? | Tired of using them | 3 (7.7%) |
|  | Irrelevant information for me and baby | 1 (2.6%) |
|  | Didn't have enough time to use them | 1 (2.6%) |
|  | Made feel anxious or worried | 1 (2.6%) |
|  | Not sure/can't remember | 1 (2.6%) |
|  | Other | 1 (2.6%) |
|  | Inaccurate information | 0 (0.0%) |
| Influence to choose the Labour and birth apps | Recommendation by friend or family | 15 (38.5%) |
|  | Cost | 11 (28.2%) |
|  | Recommendation by health care provider | 7 (17.9%) |
|  | Operating system | 7 (17.9%) |
|  | Language used | 5 (12.8%) |
|  | Other | 5 (12.8%) |
| Did you check where the Labour and birth apps developers obtained the information used for the apps? | No | 26 (66.7%) |
|  | Not sure/can't remember | 4 (10.3%) |
|  | Yes, for at least one app | 3 (7.7%) |
|  | Yes, for all the apps | 0 (0.0%) |
| Have you been concerned how your personal data might be used by app developers | No, the apps do not involve uploading personal information | 14 (35.9%) |
|  | Yes, I'm somewhat concerned | 8 (20.5%) |
|  | I'm not sure | 7 (17.9%) |
|  | No, I'm not at all concerned | 4 (10.3%) |
|  | Yes, I'm very concerned | 1 (2.6%) |

Table 4: Social media usage (N=97)

| Characteristic | Variable | n (%) |
| --- | --- | --- |
| Which platform did you use for pregnancy-related information? (could select all that apply) | Facebook | 70 (72.1%) |
|  | Instagram | 49 (50.5%) |
|  | TikTok | 10 (10.3%) |
| Average use of FB/Insta/TikTok | A few times a week | 27 (27.8%) |
|  | Daily | 23 (23.7%) |
|  | Every week or so | 17 (17.5%) |
|  | Only once or twice | 10 (10.3%) |
|  | Every month or so | 8 (8.2%) |
| What did you use FB/Insta/TikTok for? | Find information about pregnancy | 50 (51.5%) |
|  | Connect with other mums-to-be | 50 (51.5%) |
|  | Look for products to help with pregnancy aliments | 44 (45.3%) |
|  | Update family and friends with progress of pregnancy | 43 (44.3%) |
|  | Pregnancy announcement | 41 (42.3%) |
|  | Post pregnancy-related information | 38 (39.1%) |
|  | Keep up to date with latest trends or information about pregnancy | 25 (25.8%) |
|  | Keep informed about the hospital and feedback from women who birthed there | 15 (15.5%) |
|  | Connect with other women having a baby at the same hospital | 7 (7.2%) |
|  | Other | 2 (2.1%) |
| Did you check where the source of information posted by others came from? | No | 35 (36.1%) |
|  | Yes, for all FB/Insta/TikTok posts | 20 (28.9%) |
|  | Yes, for at least one posts | 20 (28.9%) |
|  | Not sure/can't remember | 9 (9.3%) |
| Have you been concerned about how personal information or image will be used by FB/Insta/TikTok? | No, not at all | 36 (37.1%) |
|  | Yes, somewhat concerned | 22 (22.7%) |
|  | No, it did not involve uploading personal information or images | 13 (13.4%) |
|  | Don't know/not sure | 9 (9.3%) |
|  | Yes, very concerned | 5 (5.2%) |
| Which social media did you use for labour and birth information?  (could select all that apply) | Facebook | 70 (72.1%) |
|  | Instagram | 49 (50.5%) |
|  | TikTok | 10 (10.3%) |
| What was the average time for used for FB/Insta/TikTok for labour and birth information? | A few times a week | 13 (13.4%) |
|  | Every week or so | 11 (11.3%) |
|  | Daily | 11 (11.3%) |
|  | Only once or twice | 8 (8.2%) |
|  | Every month or so | 6 (6.2%) |
|  | Not sure/can't remember | 1 (1.0%) |
| What did you use them for? | Reading/watching other L&B stories | 44 (45.3%) |
|  | Birth announcement | 26 (26.8%) |
|  | Connect with women of similar experiences | 23 (23.7%) |
|  | Asking for advice | 18 (18.6%) |
|  | Update family and friends | 16 (16.5%) |
|  | Offer advice/support to others | 13 (13.4%) |
|  | Give tips to others on what to expect | 12 (12.4%) |
|  | Share your birth stories | 11 (11.3%) |
|  | Edit and post of when baby is born | 10 (10.3%) |
|  | TikTok birth stories | 4 (4.1%) |
|  | Edit and post being in labour | 4 (4.1%) |
| Did you worry about where the information pasted can from? | No | 27 (27.8%) |
|  | Yes, for at least one | 12 (12.4%) |
|  | Yes, for all FB/Insta/TikTok | 7 (7.2%) |
|  | Not sure/can't remember | 4 (4.1%) |
| Have you been concerned about how your personal information might be used? | No, I'm not at all concerned | 18 (18.6%) |
|  | Yes, I'm somewhat concerned | 13 (13.4%) |
|  | No, it does not involve me uploading personal information or images | 12 (12.4%) |
|  | Don't know/not sure | 4 (4.1%) |
|  | Yes, I'm very concerned | 3 (3.1%) |

Table 5: YouTube and Video usage (N=64)

| Characteristic | Variable | n (%) |
| --- | --- | --- |
| What type of YouTube or videos did you search for in pregnancy?  (could select all that apply) | What to expect in pregnancy | 28 (43.8%) |
|  | Pregnancy exercises | 25 (39%) |
|  | Foetus development week by week | 24 (37.5%) |
|  | Product reviews | 22 (34.4%) |
|  | Tips for pregnant women | 20 (31.2%) |
|  | Pregnancy vlogs | 18 (28.1%) |
|  | Hypnobirthing/calm birthing | 15 (23.4%) |
|  | Signs & symptoms of pregnancy | 14 (21.9%) |
|  | Pregnancy must-haves | 12 (18.8%) |
|  | Food and dietary advice | 11 (17.2%) |
|  | Meditation | 7 (10.9%) |
|  | Huggies Australia | 5 (7.8%) |
|  | Having a baby in a pandemic | 4 (6.5%) |
|  | Other | 3 (4.7%) |
| What type of YouTube or videos did you search for in labour and birth?  (could select all that apply) | What to pack | 24 (37.5%) |
|  | L&B videos | 22 (34.4%) |
|  | Labour breathing techniques | 20 (29.7%) |
|  | L&B vlogs or stories | 19 (11.4%) |
|  | One born every minute | 18 (28.1%) |
|  | What to expect after birth | 16 (25%) |
|  | Stages of labour | 13 (20.3%) |
|  | Birthing online class | 12 (17.2%) |
|  | Labour inducing exercises | 11 (6.6%) |
|  | Playlist for L&B | 9 (14.1%) |
|  | Birth trauma | 1 (1.56%) |
|  | How to push during labour | 1 (1.56%) |
| How often to do you access YouTube or videos for your pregnancy or labour and birth needs? | Every month or so | 12 (18.8%) |
|  | Only once or twice | 10 (15.6%) |
|  | Every week or so | 10 (15.6%) |
|  | Daily | 8 (12.5%) |
|  | A few times a week | 7 (10.9%) |
|  | Not sure/can't remember | 2 (3.1%) |
| Have you been worried about where the YouTube or video creators obtained the information provided? | No | 31 (48.4%) |
|  | Yes, for at least one | 10 (15.6%) |
|  | Yes, for all | 5 (7.8%) |
|  | Not sure/can't remember | 3 (4.7%) |

Table 6: Website usage (N=106)

| Characteristic | Variable | n (%) |
| --- | --- | --- |
| What websites did you access for your pregnancy and labour and birth needs?  (Could select all that apply) | What to expect | 53 (50%) |
|  | Better health | 40 (37.7%) |
|  | Babycenter | 36 (34.0%) |
|  | Pregnancy, birth, baby | 25 (23.6%) |
|  | The healthy mummy | 15(14.2%) |
|  | Other | 15 (14.2%) |
|  | Babyology | 10 (9.4%) |
|  | The bubhub | 8 (7.5%) |
|  | Newborn baby | 5 (4.7%) |
|  | Cutest baby shower ideas | 2 (1.9%) |
|  | The pregnancy naturopath | 2 (1.9%) |
|  | The natural parent magazine | 1(0.9%) |
|  | The newborn studio | 1 (0.9%) |
|  | Stylish bump | 0 (0.0%) |
|  | Eve health | 0 (0.0%) |
| How often do you access the websites? | Every month or so | 24 (22.6%) |
|  | Every week or so | 24 (22.6%) |
|  | Only once or twice | 12 (11.3%) |
|  | A few times a week | 10 (9.4%) |
|  | Daily | 5 (4.7%) |
|  | Not sure/can't remember | 4 (3.8%) |
| Did you worry about the content found on websites? | No | 49 (46.2%) |
|  | Yes, for at least one | 18 (17.0%) |
|  | Not sure/can't remember | 8 (7.5%) |
|  | Yes, for all | 5 (4.7%) |

Table 7: Podcast (N=53) and online discussion group usage (N=40)

| Characteristic | Variable | n (%) |
| --- | --- | --- |
| Which Podcast did you listened to?  (could select all that apply) | Australian Birth Stories | 26 (49%) |
|  | Baby and beyond | 9 (17%) |
|  | Hello Bump | 8 (15.1%) |
|  | Other | 4 (7.5%) |
|  | The birth hour | 2 (3.8%) |
|  | Doing it at home | 2 (3.8%) |
|  | Informed pregnancy | 0 (0.0%) |
|  | 40 weeks pregnancy | 1 (1.9%) |
|  | Birth | 1 (1.9%) |
|  | Birthful | 1 (1.9%) |
|  | The push guide | 0.0% |
|  | Pregnancy podcast | 0.0% |
|  | Birth kweens | 0.0% |
|  | Indie birth | 0.0% |
| How much time do you spend listening to Podcast? | Daily | 29 (54.7%) |
|  | Only once or twice | 14 (26.4%) |
|  | Every week or so | 10 (18.9%) |
|  | A few times a week | 3 (5.7%) |
|  | Not sure/can't remember | 3 (5.7%) |
|  | Every month or so | 2 (3.8%) |
| Did you ever worry about the content posted but Podcast creators | No | 21 (39.6%) |
|  | Yes, for at least one | 7 (13.2%) |
|  | Not sure/can't remember | 3 (5.7%) |
|  | Yes, for all | 3 (5.7%) |
| What activities do you engage in the online discussion groups? | Read other people's posts | 36 (37.5%) |
|  | Post my own questions | 24 (25.0%) |
|  | Post about my own experiences | 21 (21.9%) |
|  | Post advice to others | 15 (15.6%) |
| How much time do you spend on online discussion groups? | Every month or so | 11 (27.5%) |
|  | Only once or twice | 9 (22.5%) |
|  | Every week or so | 8 (20%) |
|  | A few times a week | 6 (15%) |
|  | Daily | 5 (12.5%) |
|  | Not sure/can't remember | 1 (2.5%) |
